# The house as a mind

**DOI:** 10.12688/f1000research.130471.1

**Published:** 2023-03-20

**Authors:** Clive Gamble

**Affiliations:** 1 Centre for the Archaeology of Human Origins, University of Southampton, Southampton, England, SO171BF, UK

**Keywords:** Deep history, containers, Neocortex, cerebellum, houses, Palaeolithic, human evolution

## Abstract

Palaeoanthropologists and evolutionary psychologists have successfully used the increasing size of the brain during human evolution to infer cognitive and social outcomes. Archaeologists have applied similar reasoning to the development of technology in deep history. This paper goes beyond these approaches by considering the house as a metaphor for the structure of hominin minds. It is argued that the study of the mind in deep history requires, (1) a recognition that mind is distributed between bodies, brains, and the world. The implications are examined through a magnetic resonance imaging (MRI) study (that unwraps the cerebellum and which suggests that folding rather than cortex size may be more important for understanding cognition.; (2) unmasking the ingrained container- *habitus* that has been used to describe and investigate minds either in the present or deep past. This bias is explored by entering the eccentric house-mind of Sir John Soane (1753-1837) with its many compartments, paintings, and antiquities; and (3) an exploration of alternative embodied metaphors to enable archaeologists to study distributed mind in deep history. The metaphor ARCHITECTURE WITHOUT WALLS is discussed and briefly compared to the evidence for ‘houses’ in the Middle and Upper Pleistocene. The evidence indicates that hominins have always had complex, distributed minds but only recently in our deep history did we come to think predominantly through and with artificial containers such as houses. Late in human history these constructions became a common-sense *habitus* that expressed and fashioned our cognitive experience of the world.

## Introduction

Houses have agency because, as Alfred Gell declared, the house is a body for the body ( Gell, 1998: 252-3). Houses act as bodies because, like bodies, they are containers with surfaces, entrances, and exits. They have organs of sense and expression; gaudy skins, spyholes to peer through and voices which reverberate through the night. Gell concluded that, “To enter a house is to enter a mind, a sensibility” (ibid: 253). The question is, what kind of mind?

In this paper I examine three metaphors of containment and their implications for cognition and mentalizing in deep history. These are the embodied metaphors of THE MIND’S EYE, THE WALLS HAVE EARS, and THE DISTRIBUTED MIND. I also examine the house as a practical metaphor of embodiment using a free-to-visit example, Sir John Soane’s Museum in London. I have two aims. First, to unmask a container- *habitus* in the study of contemporary and historic humanity. Second, to investigate the distributed mind through a more appropriate practical metaphor, ARCHITECTURE WITHOUT WALLS, for a Pleistocene-scale deep history. With this perspective the house, as an object of archaeological enquiry, provides an opportunity to understand how cognition evolved, not simply as a function of larger brains but through the bodily experience of containment.

Houses were once, like minds, private, locked places. Access was by invitation only and a physical visit was needed. No longer. The internet allows us via house selling sites [Rightmove (rightmove.co.uk/), On the Market ( onthemarket.com/), and Zoopla ( zoopla.com/)] to enter thousands of properties at the click of a mouse. Like field-archaeologists, we can explore their contents, measure their floorplans and infer the owners hopes, dreams, and economic status, even give them a psychological profile. If houses are, as Gell suggests, minds, then there has never in humanity’s history been such open access and so many minds to explore. And all without a person visible. We understand minds in this online estate through the agency of things and our cognitive belief that houses, like minds, contain them. And it is this container- *habitus* which shapes our world and our past.

### THE MIND’S EYE

To cross the threshold of Sir John Soane’s Museum in Lincoln’s Inn Fields, London, is to enter the mind of an architect and avid collector of classical antiquities ( soane.org/). Soane zealously curated every detail of his career in an attempt, according to Sophie Thomas (2018: 131), “to fortify, document, and defend his life”. She correctly describes him as an archivist of the self. Soane’s house-museum is a biographical bundle, frozen in time. Its warren of rooms and spaces creates a house within a house into which his stuff was poured, then sealed by his will. But if we only regard his museum as a time-capsule of a life lived between 1753 and 1837, we miss the point of how Soane’s houses and their contents work as a mind.

**Figure 1.**
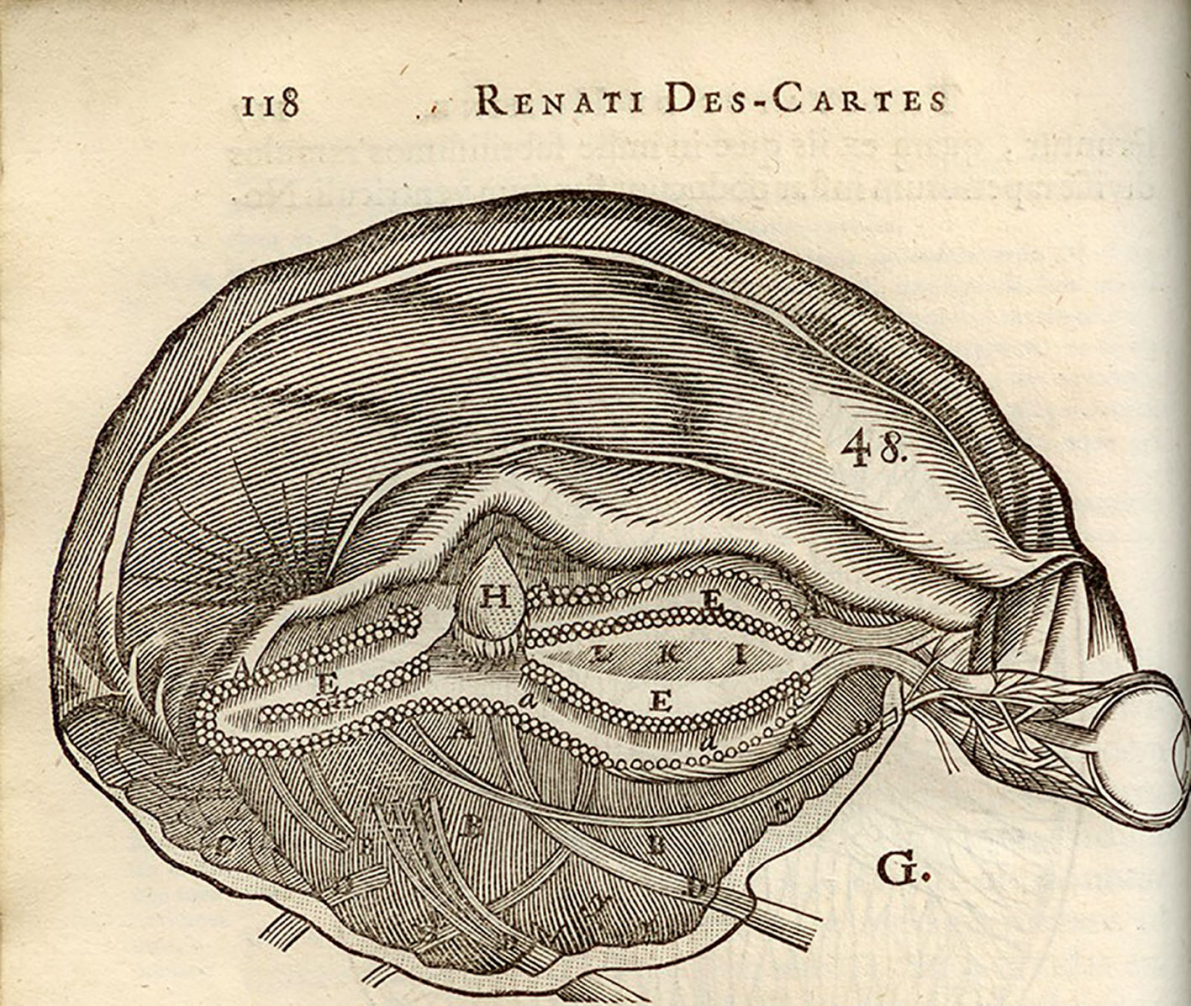
The central position of the pineal gland, H, contained deep within the brain. The image appeared in Descartes’s Treatise of Man published posthumously in 1662.

Soane’s houses are containers which resemble the disembodied mind inherited from René Descartes (1596-1650) with its interior spaces and machine-like pipes and pumps regulated by an internal overseer; a social architecture revealed by bisecting the brain ( Figure 1).

At the centre of this boxed-in-brain sits the pineal gland, Descartes’s seat of reason and ‘common’ sense, but now caricatured by Daniel Dennett (1991: 106-7) as neither the turnstile of consciousness nor the Oval Office of the brain. Nonetheless, its central, enclosed position within the brain produced a powerful metaphor to express this containment, THE MIND’S EYE ( Table 1), which Descartes used in the *Regulae*, published between 1619 and 1628.

**Table 1.** Descartes, *Rules for the direction of the mind* ( Murdoch *et al.,* 1985). These rules still underpin the methods used by most scientists.

**Rule 5** ‘The whole method consists entirely in the ordering and arranging of the objects on which we must concentrate our mind’s eye if we are to discover some truth. We shall be following this method exactly if we first reduce complicated and obscure propositions step by step to simpler ones, and then, starting with the intuition of the simplest ones of all, try to ascend through the same steps to a knowledge of all the rest’, **Rule 9** ‘We must concentrate our mind’s eye totally upon the most insignificant and easiest of matters, and dwell on them long enough to acquire the habit of intuiting the truth distinctly and clearly.’ ( Murdoch *et al.,* 1985: 20 and 33)

The pineal gland and THE MIND’S EYE point to Descartes’s unspoken reliance on containers to express his cognitive system metaphorically. As summarised by George Lakoff and Mark Johnson (1999: 395) this has handed down to us three embodied metaphors which still dominate the description of how minds are understood. These are, THE MIND IS A CONTAINER OF IDEAS, IDEAS ARE OBJECTS, and KNOWING IS SEEING. And these are not just semantic metaphors. Sir John Soane’s Museum is also a practical container metaphor, equivalent to Chris Tilley’s (1999) concrete metaphor, expressed in material form ( Gamble, 2023 in press). It is an example of a house-mind that is a compartmentalized, practical metaphor containing ideas, objects, and knowledge.

Descartes and most who followed him worked in what I refer to as a world of container- *habitus*; an unrecognized bias to think predominantly in terms of the bodily experience of containment, enclosure, and compartmentalization. Tim Ingold with customary perspicacity has unmasked this dominant cognitive trait,

“This experience of containment influences our thinking about what it means to inhabit a world to an extent that even psychologists and philosophers, who are tasked with the investigation of such matters, are ill prepared to recognise” ( Ingold, 2015: 41).

Archaeologists can be added to Ingold’s list of those who use, but do not recognise the formative influence of containment on cognition. However, archaeologists are not usually expected to do humanity’s thinking about what it means to inhabit a world. That has been left to those disciplines like anthropology and sociology which can speak with their subjects, or to historians who have access to minds through texts and archives. Rather, our primary task has always been to understand the history of inhabiting through things.

This role needs to change. Archaeologists can access hominin minds through metaphors that are older than language which shapes cognition with its semantic metaphors. We do this through practical metaphors, stuff. But as I have shown elsewhere ( Gamble, 2007) the stuff which contains humanity developed and increased during deep history. The Neolithic sees an exponential rise in containers among them villages of mud-brick houses, graves in cemeteries, pots, stone bowls, textiles, bags, baskets, and more people ( Gamble, 2023 in press). An explosion that outstrips arguments that selective preservation of perishable materials has skewed the evidence. Today we inhabit a hyper-container world where we live, in James Gibson’s words, ‘boxed-up lives’( 1979: 203). Archaeologists reflect this unconscious, culturally inherited container- *habitus* by tracing the origins of modernity to the Neolithic explosion of container artefacts. This is apparent in the prehistories of Gordon Childe (1942) and Colin Renfrew (1973, 1996) while the same container- *habitus* is found throughout Yuvral Harari’s (2014) global history. The container- *habitus* in these narratives may be camouflaged by economic and symbolic smokescreens, but a puff of wind reveals the origin of our ‘boxed-up lives’ to be in ‘boxed-up prehistoric lives’.

None of this is surprising. If we lift the lid on the Neolithic box of deep history it smells of home. This is not the case with the Palaeolithic box. Not because of smaller brains and the presence of hyenas but because there is much less stuff-that-contains inside its box. It is for us a strange world where our familiar cognitive props, those practical metaphors of containment, elude us.

### THE WALLS HAVE EARS

**Figure 2.**
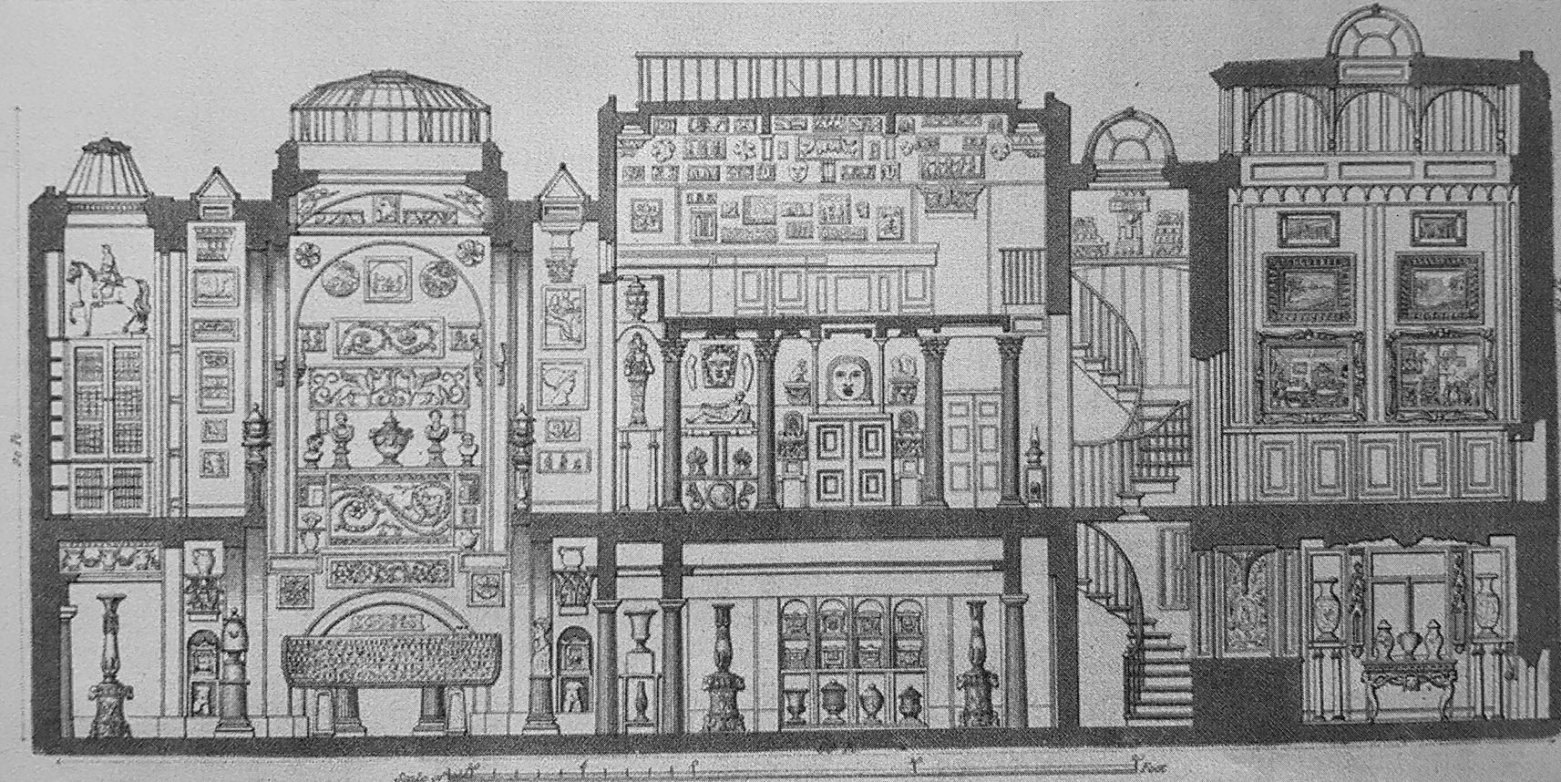
Soane’s house-mind-museum. The Crypt with Seti’s sarcophagus is on the left, the picture gallery on the right ( Soane, 1835-1836: Plate XXV).

Having recognised the container- *habitus* what can be done about it? In Sir John Soane’s house-mind-museum we shouldn’t be deceived by the solid walls which partition the spaces room by room, floor by floor; from the sarcophagus of Seti I ^st^ in the basement to his architectural drawings for the Bank of England in the attic ( Figure 2). Such rigid compartmentalization encourages us to look for rational connections between spaces and objects and by deduction the mind that arranged them.

Instead, if we collapse the internal boundaries we can approach Soane’s house-mind like the gyrifications of the cerebral cortex; where precise folding and wrapping packs a brain, like a parachute, into its brain-case. Scanning by magnetic resonance imaging (MRI) now reveals the extent of this folding for the hindbrain, the cerebellum. Compared to the neocortex the cerebellum is small, a tightly folded bundle of neuronal tissue with approximately 10 percent of the volume of the brain’s cortex. When unwrapped using MRI it becomes a two-dimensional, wafer-thin strip measuring 10cms by an astonishing 1metre ( Sereno *et al.*, 2020). The unwrapped cerebellum now grows exponentially in size achieving 78 percent of the total surface area of the neocortex. By comparison the macaque’s cerebellum when unwrapped achieves only 33 percent.

Human evolution, it appears, has favoured growth in both the neocortex and the cerebellum. Folding, the process of gyrification, is therefore as important as relative brain size, a figure driven principally by the neocortex. The folding of the folia also provides another answer to the question, what kind of mind?

A tightly swaddled cerebellum brings different areas of sensation into contact in a kaleidoscopic fashion; the example given by Sereno (2020) is a bit of lip found next to a chunk of the shoulder. As a result, the idea that the cerebellum primarily controls balance and movement needs to be augmented. In Sereno’s view it contributes not only to our five senses but also to pain, thought, and emotion. Its compact versatility contrasts with the neocortex where distinct lobes are associated with particular senses; vision in the occipital, sound in the temporal and so on ( Carter, 2003).

**Figure 3.**
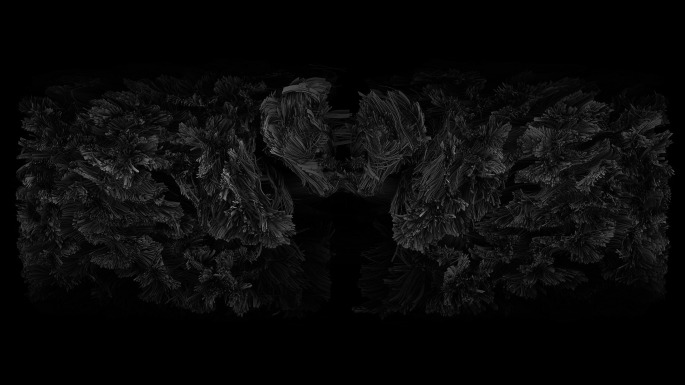
An MRI image from the Wellcome Collection of an unfolded adult brain. The image shows a Mercator transformation of the 3D space embedding a dense human brain tractography. The unfolding was performed from a ventral point of view and shows the arrival of white matter fibres to the neocortex. The cerebellum is at the top of the image at the midline. Immediately to the sides are the left and right temporal lobes, and the interhemispheric margin appears at the left and right borders of the image. Compiled by Katja Heuer and Roberto Toro https://wellcomecollection.org/works/trdzdrwk CC BY 4.0.

Clearly there is much for neuro-imagers still to discover. What I take from these MRI images ( Figure 3) is not just the physical folding of the brain, its gyrifications, but what folding affords for our metaphors of cognition. These MRI images provide a different embodied metaphor for the minds of deep history; one where wrapping and folding rather than size alone created relations and associations between people, stuff, and the world.

For the moment let’s investigate the implications for understanding Sir John Soane’s Museum, and by extension any house. First, the house as a mind is a spatial suite of folded surfaces. It is never rigidly compartmentalized. Second, different times, spaces, materials, and people are brought into association through those folds. Bruno Latour (2005: 201) observes that time is always folded. He builds on Michel Serres’s ( Serres and Latour, 1995) use of a crumpled handkerchief to show how a single surface of space and time can be changed by rubbing different spaces together. Such folding creates relations between things which are not always amenable to rational analysis. Time and space are best understood, like the unwrapped MRI brains ( Figure 3), as folded folia. And from an archaeological perspective those folia wrap stuff.

**Figure 4.**
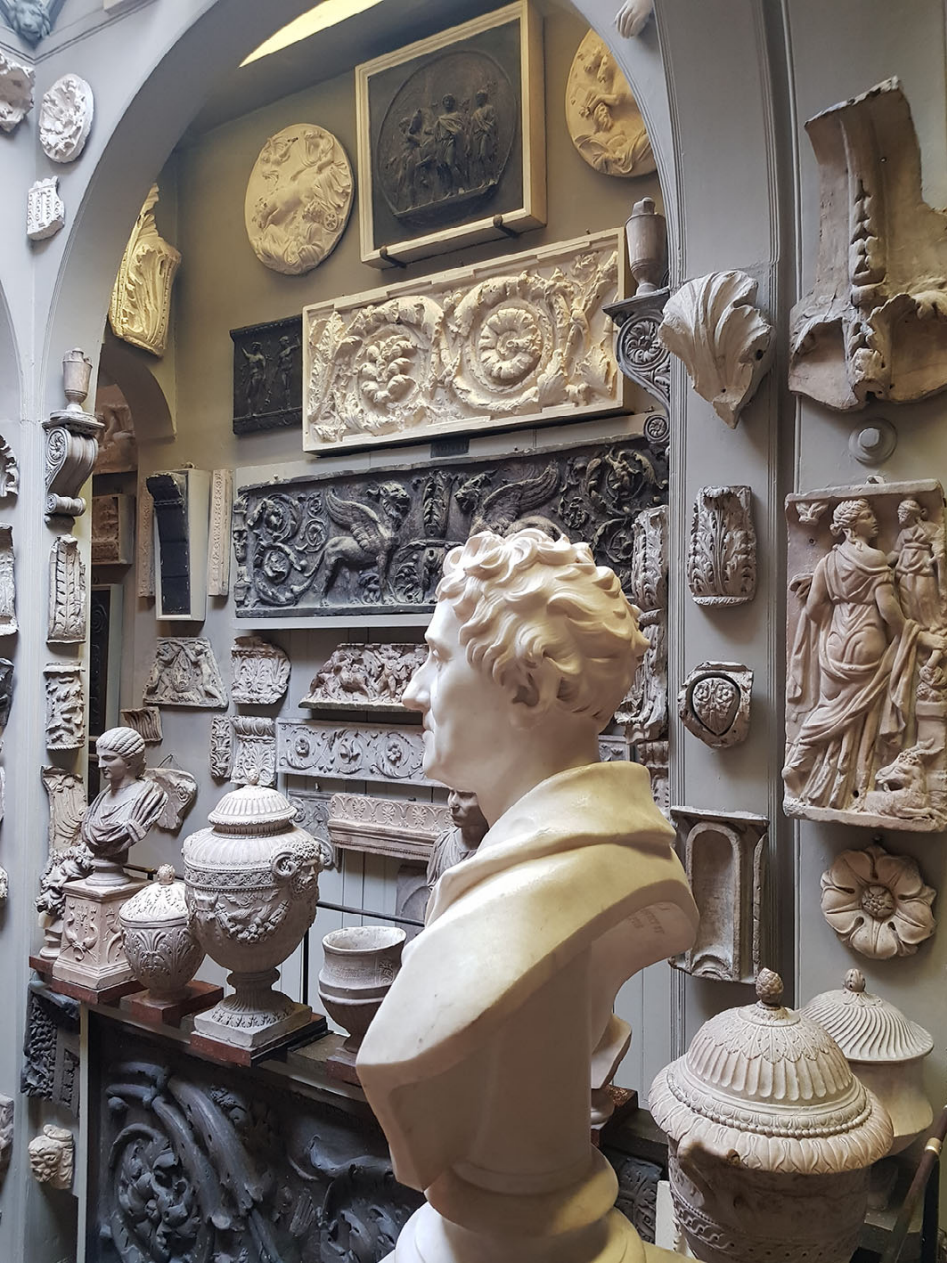
Soane’s classical bust draws the eye, sitting like the pineal gland in Figure 1 at the centre of his house-mind (see Soane, 1835-1836: Plate XXVIII). Photo Clive Gamble.

Soane arranged his collections in the spaces he designed to unite the three arts of architecture, painting and sculpture and as ‘studies for my own mind’ (1835-6:vii). His marble bust, centrally positioned, is in visual contact with many classical antiques and it overlooks Seti’s sarcophagus in the basement ( Figure 4). The millennia of time are folded within the space of his fixed gaze and the ambulatory perception of the visitor. Chronology did not interest him. His museum reminds me of John Phillips (1860: 51), President of the Geological Society, who declared that geological time “eluded the grasp of the imagination”. But he also described it as folded with this example: a gravel quarry in Oxfordshire, five metres deep, with mammoth bones at its base and the footprints of Charles I ^st^ fleeing the Parliamentary army at the top. In a single stratified section recent and deep history touched, concertinaed into each other ( Gamble, 2021: 132). Time and space are also wrapped and folded in Soane’s Museum, from the depths of Seti’s sarcophagus to his own portrait bust ( Figure 4).

Soane’s museum is a projection of the MRI kaleidoscope of the cerebellum as described by Serreno. Lips touch shoulders, compassion nestles against pain. Rather than a MIND’S EYE organizing everything it is instead a house where WALLS HAVE EARS.

This kaleidoscopic folding is on show in Soane’s Picture Room; a small space crammed with paintings by Canaletto, Turner, and many others. The room is deceptive. It is a skin wrapped within a skin. The paintings are hung on folding doors, or planes, that open to reveal another layer on the reverse side. At one level this is a practical solution for a larger-than-the-space-available collection. But these first and second skins also fold time: in this case the fifteen ink and wash drawings by Giovanni Battista Piranesi (1720-1778) showing the ruined Greek temples at Paestum, in Italy ( Thornton and Dorey, 1992: 41). These are hung alongside William Hogarth’s (1697-1764) eight paintings of *A Rake’s Progress* with its different take on decline and decay. Piranesi shows the collapse of civilised glory in the face of time, while for Hogarth it’s the rapid ruination of a young man, Tom Rakewell; the history of a civilisation contrasted with a single biography. Time and space are always wrapped, their folia folded to make new patterns, fresh associations.

### MIND IS DISTRIBUTED

The ingrained container- *habitus* of many archaeologists studying cognitive evolution led Chris Gosden (2010: 40) to announce the death of such a mind to be replaced with the indivisible trinity of bodies, brains, and world ( DeMarrais *et al.,* 2004; Knappett, 2005; Dunbar *et al.,* 2010; Conneller, 2011; Malafouris, 2013; Spikins, 2015). The mind that emerges from this trinity extends beyond our self-contained borders; our skins, clothes, kin, and houses, so that as Andy Clark (1997; Clark and Chalmers, 1998) states, “everything leaks”. As a result, cognition is porous and the agency arising from mentalizing walks a three-way street between brains, stuff, and the world we inhabit.

This understanding of cognition is expressed in the embodied metaphor MIND IS DISTRIBUTED. If mind is extended in this way, then the concept breaks free from the familiar container- *habitus* that guides how we think we think. Boundaries are replaced by flows and the solid partitions between categories such as reason and emotion collapse to resemble instead the folia in the tightly folded cerebellum ( Figure 3).

Soane’s Museum is a mind distributed in time and through spaces. Thomas (2018) recounts how Soane extended himself beyond his death. He placed three time-capsules in his house and instructed his trustees to open them in 1866, 1886, and 1896. The contents of these sealed locations were regarded by the curators as a disappointment; among them newspapers, lottery tickets, stubs of cheque-books, bills, theatre tickets, notes from the spies he hired to follow his eldest son, and a pair of false teeth. In short no hidden masterpieces or even the key to the mysteries of the house and its contents. Simply the ephemera of a life lived. Thomas (2018: 138) draws a parallel with the 610 cardboard storage boxes that Andy Warhol filled on a daily basis with the mundane stuff that crossed his desk ( https://www.warhol.org/timecapsule/andy-warhols-time-capsule-21/). These containers may be sealed like any tomb so that at first sight Warhol’s boxes and Soane’s time capsules pack neatly into the container- *habitus* to produce a familiar mind arising from ‘boxed-up lives’. But that is because we are trained to see the walls of any container as impermeable; skin, for example, contains our inner selves, while clothes present outward identities; a case of ‘there’s the essential Warhol in a box’, or ‘Let’s find the real Soane in a locked drawer’. The distributed ‘Warhol’ and ‘Soane’ are not, however, neatly bounded entities with discrete minds. Although the artist and the architect both used containers to box their lives, they could not obscure the flows of agency between the trinity of brains, stuff, and the world. Instead, what they created transcends such embodied experience.

### ARCHITECTURE WITHOUT WALLS

In his influential study of the prehistoric mind Steven Mithen (1996: 65-72) uses the container metaphor of church architecture to explain the process. Around the central nave of generalized intelligence were built four side chapels of specialized knowledge; technical, linguistic, social, and natural history. At some point the separate knowledge in these chapels was integrated by the evolution of cognitive fluidity. The result was a disembodied, super intelligence that eventually would write Descartes’s *Regulae* and drive the driverless car.

From the perspective of the distributed mind, I would offer a different metaphor, an ARCHITECTURE WITHOUT WALLS, to counterbalance the slowly evolving cathedral. Houses serve as proxies for minds not because of what they contain, or how many rooms they have, but how they mediate the flows of agency between brains, bodies, and the world. Houses, like brains, can vary in size and complexity from Diocletian’s palace to an igloo. Brain size increases during hominin evolution and particularly during the Middle Pleistocene, 800,000 – 125,000 years ago ( Gamble *et al.,* 2014; Pope *et al.*, 2018). Philip Rightmire (2004) identifies an increase of c. 20 per cent in brain size and encephalisation quotients between 0.6-0.2 million years ago. This significant growth in such an energetically expensive tissue ( Aiello and Wheeler, 1995) is not however matched by a comparable shift in stone, or any other hominin technology, and certainly not in containers. The changes are instead to be found in the persistent use of places and landscapes ( Shaw *et al.,* 2016) that has been described by Matt Pope (2018) as crossing a threshold in hominin evolution.

Container artefacts, most notably hearths ( Alperson-Afil and Goren-Inbar, 2010; Gowlett, 2010, 2016; Karkanas *et al.,* 2007; Zhou *et al.,* 2012; MacDonald *et al.*, 2021), are present both before and during this time of increasing brain size. But houses are not ( Kolen, 1999). There have, of course, been claims for substantial structures. At Olduvai Gorge, site DK Level 3, Mary Leakey (1971: Fig. 7) interpreted a circular agglomeration of stones with a maximum diameter of 4.3 metres as the base of a hut. Richard Potts (1988), however, interprets this 1.9 million year old accumulation as a stone cache.

**Figure 5.**
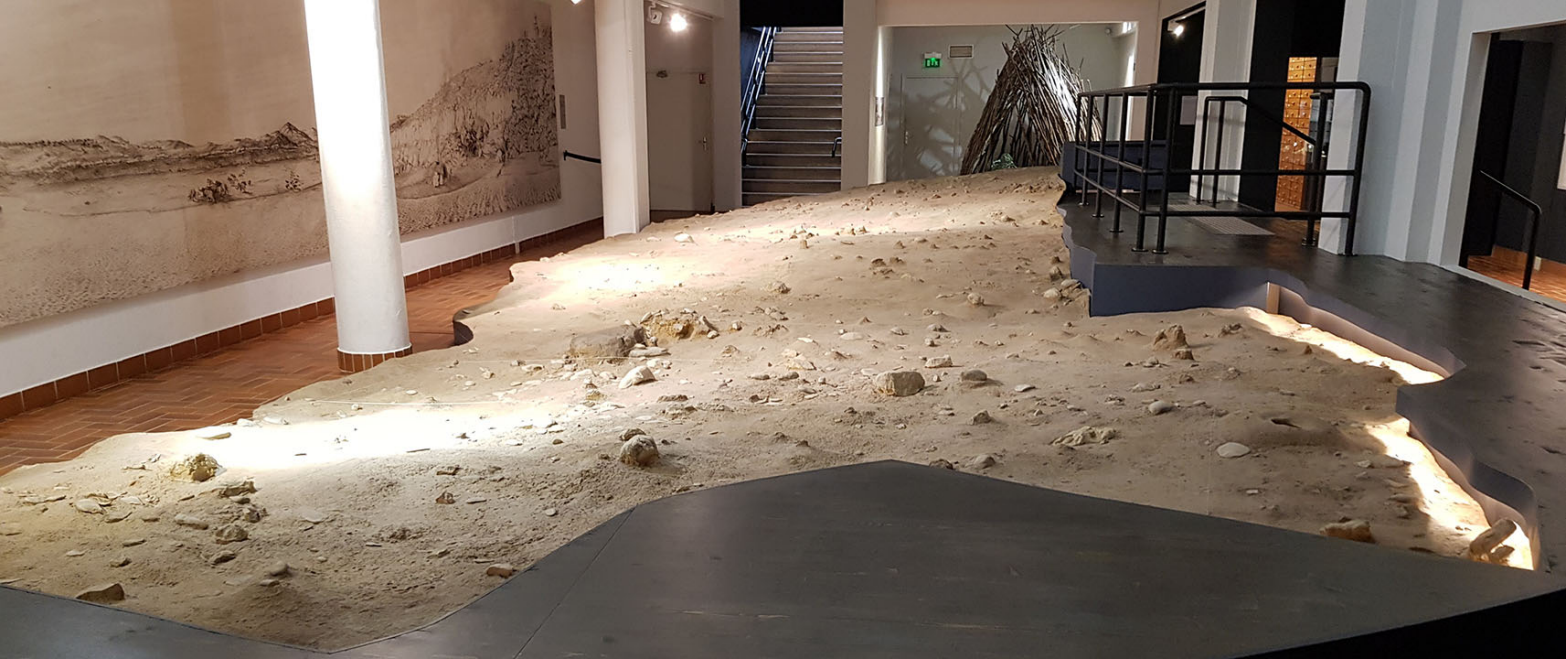
The hut displayed in the Musée Terra Amata, Nice, dated to MIS 10,400–380,000 years ago. 21 living floors with huts ranging in length from 8 to 15 metres have been identified (de Lumley, 2009) photo Clive Gamble.

From the Middle Pleistocene of Europe, the multiple long huts with hearths at the coastal locale of Terra Amata, France ( de Lumley and Boone, 1976; de Lumley, 2009) ( Figure 5) have not stood up to detailed scrutiny ( Villa, 1976-1977, 1982). The Bilzingsleben huts in Germany ( Mania, 1990, 1991) are part of a fan deposit subsequently criss-crossed by small geological faults. The geomorphological setting questions the claims for good preservation that are essential to the interpretation of dwellings ( Gamble, 1999: 153-163). On the other hand, Middle Pleistocene locales with exceptional preservation such as Schöningen, Germany ( Conard *et al.,* 2015) and Boxgrove, England ( Pope *et al.,* 2020) lack evidence such as post-holes for structures. As Jan Kolen (1999: 162) concluded more than 20 years ago, the evidence from Europe is most economically interpreted as centrifugally produced living structures rather than architecture. A pattern that results from the activities of bodies using space. An example of this patterning is the ‘cabin’ inside Lazaret Cave, France ( de Lumley *et al.,* 2004).

**Figure 6.**
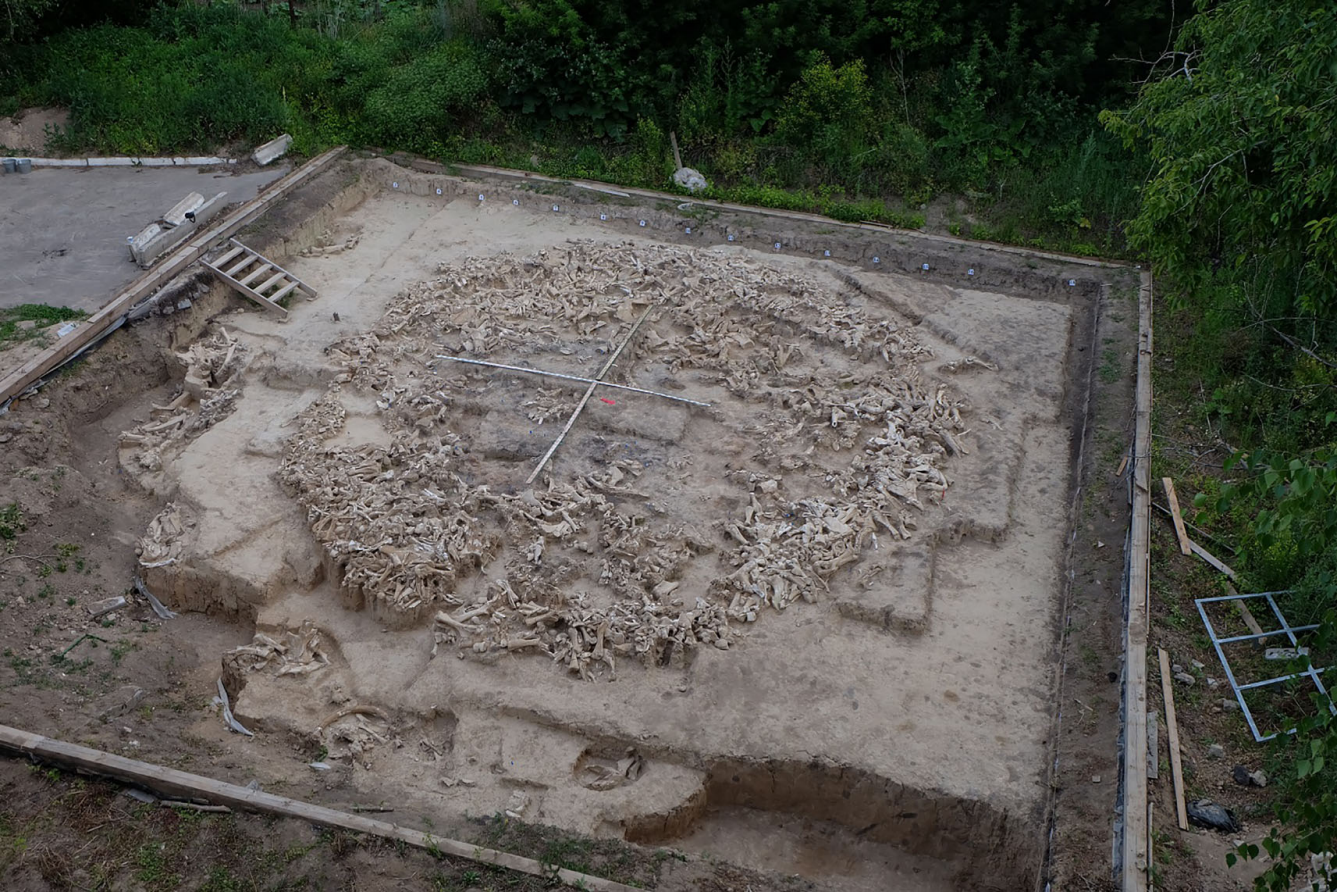
Excavations of the mammoth bone structure at Kostenki 11 level !a in 2017. The structure is 12.5 metres in diameter and is the third found at K11. It is dated to 25,000 years ago ( Pryor *et al.*, 2020). Photo courtesy of Alexander E. Dudin.

Kolen also observed that the first 10,000 years of the European Upper Palaeolithic (c. 40-30,000 years ago) mostly lacks durable architecture and unambiguous burials with grave goods. These start no earlier than the Pavlovian (32-30,000 years ago) of the Czech Republic and extend eastwards into Ukraine and Russia. Even so, the evidence for huts and houses remains contentious. For example, at Kostenki 11, Russia ( Figure 6) recent excavations of a mammoth bone structure have shown, that whatever else it is, it is not a house ( Pryor *et al.,* 2020).

This brief survey of artificial structures in deep history reminds us that the most potent practical metaphor for a mind, the house, is absent. The evidence, of course, is biased towards Europe. Houses older than 30,000 years may yet be found in Africa and Asia. But by comparison hearths, that form such a significant feature in many houses, are found throughout the Old World in the Lower and Middle Pleistocene ( Gowlett, 2016).

**Figure 7.**
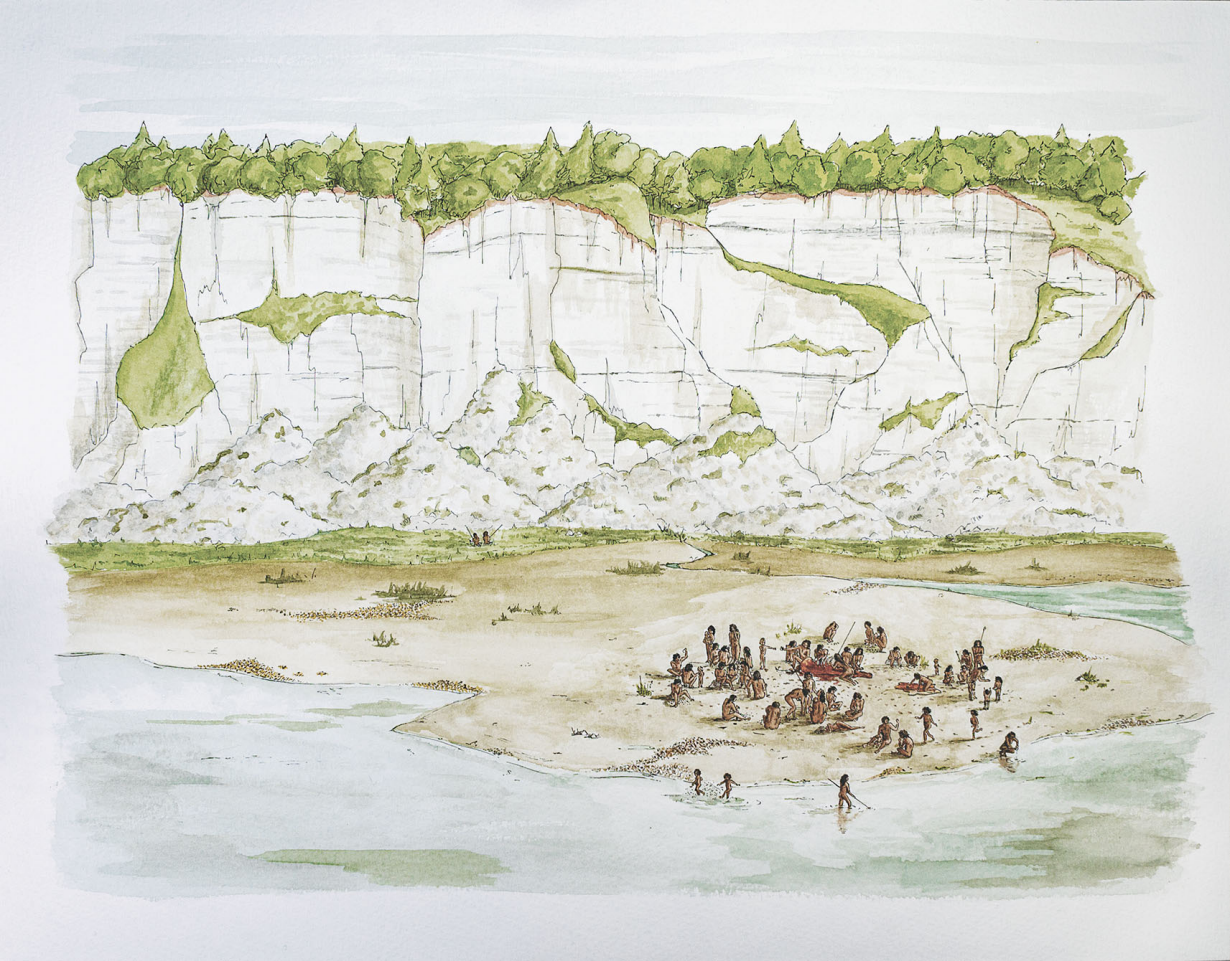
The coastal marshlands at Boxgrove. Artist Lauren Gibson sets the scene of the horse butchery site, GTP-17. A performance space is created by the ring of people involved in an activity that took place one day, half a million years ago. Reproduced with the permission of Lauren Gibson and UCL Institute of Archaeology and © UCL 2020. CC BY-NC-ND licence.

We are indeed confronted by an ARCHITECTURE WITHOUT WALLS where spatial patterning exists but without being artificially contained. A moment’s reflection suggests this is unsurprising. Searching for archaeological evidence for houses betrays our container- *habitus* when it comes to organising and investigating the past. But hominins have always authored spaces to enact the varied performances of social life. One such example is the horse butchery locale at Boxgrove, 500,000 years ago. Matt Pope *et al.* (2020, Pope, 2020) have shown how the evidence reveals the minute-by-minute movement and activities of a tight-knit group; a community of people, young and old, working together in a co-operative and highly social way. From the flint and bone evidence they propose a group of at least 30-40 people ( Figure 7).

The patterning of the artefacts in front of the Boxgrove cliffs suggests that people formed a ring around the horse carcass, an area of about 90 square metres. Here was a temporary container of social life, an ARCHITECTURE WITHOUT WALLS formed by the participants in a gathering. The architecture of this event needed neither partitions nor hearths and certainly none of Gell’s gaudy skins that dominate Soane’s Museum. It is an image of the distributed mind, recovered by archaeologists from the fragments of deep history. Hominins have always gathered. Social life has always been performed within porous containers as bodies shifted in the ring to get a better view.

## Conclusion: beyond big brains

Hominin minds have always existed irrespective of either the size of the neo-cortex in their skulls or if they built and lived in houses. In this paper I have argued that linking significant changes in hominin cognition such as language, planning, and memory to the evolution of big brains is an instance of our own boxed-up thinking applied to hominin evolution. We need to move beyond a deep history conditioned by an unrecognised container- *habitus* where minds are compartmentalized by the artefacts we think with and through, without thinking.

A first step is to recognise hominin cognition as distributed between stuff, brains, and the world. The stuff I have examined here are houses and I started with Gell’s statement that to enter a house is to enter a mind, a sensibility. That is patently the case with the houses of Mesolithic Lepenski Vir ( Borić, 2016), the rich house ethnographies of Africa ( Denyer, 1978) and North America ( Morgan, 1881), the ephemera of nomad tents ( Cribb, 1991: Chapter 7), and the properties currently for sale on Rightmove and Zoopla. Sir John Soane’s Museum is an eccentric example but makes Gell’s (1998: 251-8) case as effectively as his chosen example of a Maori meeting house.

The lack of credible houses before 30,000 years ago does not signify there were no minds. The evolution of larger more complex houses since then does not suggest a concomitant increase in the cognitive power of those who built and lived in them. So why should the evolution of bigger brains necessarily suggest, like building a cathedral, either more complex or different minds?

Brain size can be used to predict growth in an individual hominin’s network of relations, while the evolution of language is part of the story of a social brain hypothesis where the benefits of social life selected for encephalisation ( Dunbar, 1998; Dunbar *et al.,* 2010; Gamble *et al.,* 2014). But as James Cole (2015) has pointed out, increasing brain size among hominins reveals possibilities for the cognitive underpinnings of social life, not what was realised. Only archaeology can do that, because only archaeology can access the varied scales of time and place to write the narrative of humanity. Brain size is one factor and by considering it we have moved forward our understanding of hominin mentalising as an evolutionary process ( Gamble *et al.,* 2011; Gowlett *et al.,* 2012). But brain size now needs to be complemented by the folding of the folia and the associations that they allow and which we are only just beginning to appreciate.

Deep history has never been a foreign country, the Originsland I have written about elsewhere ( Gamble, 2007), unless it is viewed through the lens of the container- *habitus.* What binds us to deep history is a distributed mind and an ARCHITECTURE WITHOUT WALLS that guides social life. That is not to say that change didn’t occur. It did. At some point in humanity’s story containers, as exemplified by houses, came to do our thinking for us. Understanding why that happened remains one of the great challenges for deep history. And to answer that we need to unwrap the brain.

## Data Availability

No data are associated with this article.
