# Supplementary material for: The house as a mind
Source: F1000Res. 2023 Mar 20;12:301. [Version 1] doi: 10.12688/f1000research.130471.1 (PMC10442590; doi:10.12688/f1000research.130471.1)
Supplement: Peer review history

# Peer review: Reviewer response for version 1

**DOI:** 10.5256/f1000research.143232.r180841

**Published:** 2023-08-21

**Authors:** Andy Jones

**Affiliations:** 1 Department of Archaeology and Classical Studies, Stockholm University, Stockholm, Stockholm County, Sweden

This paper is vintage Gamble! Erudition and Big ideas covering large expanses of prehistory, presenting us with a whole new vision of the Palaeolithic and later periods. This paper is sure to be influential and I recommend that it is indexed verbatim (i.e. no corrections). 

 There are a few things I think the author could think about for improving the paper (for future development):

1. I was curious that the concept of 'Distributed Minds' was used without mentioning one of Gell's prime examples: the Maori meeting house. This seems to be relevant to the argument presented here. 
2. Folding. It might be worth looking at Gavin Lucas' Making Time book which develops the scrumpled tissue metaphor for time. Also worth looking at Gilles Deleuze on the fold - this is where Latour stole the idea from!
3. The critique of archaeologists as possessing the container-habitus is well taken, but I wondered how this might relate to the Wengrow-Graeber 'Dawn of Everything' thesis? Here Wengrow and Graeber seem to be offering a more fluid understanding of early societies from the Palaeolithic to Neolithic. It might be worth addressing in future. Does this connote an 'architecture without walls'?
4. It is a shame that the story ends in the upper Palaeolithic. Continuing the story into the Mesolithic and Neolithic will probably pay dividends - particularly as we encounter a proliferation of evidence for houses in these later periods. It might be worth looking into Ian Hodder's concept of 'History houses' at Catal Hoyuk in this regard. Also Richard Bradley's recent publications on houses. 
5. Finally, how do we regard caves? Surely these are containers too? The thesis developed here helps us think afresh about Cave art, and the (relatively scant) evidence for settlement and burial in Cave contexts. Does the container-habitus begin with the cave, and become transferred to built architecture at a later date? Was the architecture without walls first generated in places with natural walls (making the walls redundant), allowing for distributed minds to unfold outside the cave?

Is the topic of the opinion article discussed accurately in the context of the current literature?

Yes

Are arguments sufficiently supported by evidence from the published literature?

Yes

Are all factual statements correct and adequately supported by citations?

Yes

Are the conclusions drawn balanced and justified on the basis of the presented arguments?

Yes

Reviewer Expertise:

Prehistoric art and material culture (later prehistoric and ethnographic).

I confirm that I have read this submission and believe that I have an appropriate level of expertise to confirm that it is of an acceptable scientific standard.

# Peer review: Reviewer response for version 1

**DOI:** 10.5256/f1000research.143232.r168519

**Published:** 2023-05-03

**Authors:** John Chapman

**Affiliations:** 1 Department of Archaeology, Durham University, Durham, England, UK

Clive Gamble’s opinion piece portrays a dazzling combination of disparate ideas enlisted to make the case for closer links between recent developments in the science of the brain and developments ‘out there’ in the world, whether concerning time, buildings, landscapes or social structures. We cannot but admire the range of thoughts that Gamble cites in support of his key metaphor that the folding of folia in the cerebellum stands for growing networks, increasing social complexity and developing bodily skills in the Palaeolithic. Interleaved with Latour’s notion that time is always folded and Gell’s idea that “to enter a house is to enter a mind”, we have Gosden’s trinity of bodies, brains and the world connected by the flow of agency, as well as Gamble’s own research on the expanding use of containers – whether settlements, houses or even minds. These thoughts give rise to other ideas, principally in the form of metaphors. Indeed, this opinion piece is brimming over with metaphors, which range from six separate if interlinked embodied metaphors (" *the mind’s eye*"; " *the walls have ears*"; " *the distributed mind*"; " *the mind is a container of ideas*"; " *ideas are objects*"; " *knowing is seeing*") to two metaphors for hunter-gatherer settlement – Mithen’s church architecture and the author’s " *architecture without walls*" – and finally a metaphor for later developments – the " *container-*habitus".  This shimmering metaphorical surface shines and sparkles, working its magic on the reader much in the form of Gell’s dazzling canoe-prow that engendered better exchange rates for Melanesian mariners. There is time and space here to scrutinize more closely only the change from one metaphor to another – how the Neolithic container- *habitus* developed out of, or in contradistinction to, the Palaeolithic and Mesolithic ‘architecture without walls’. This is the great container shift which Gamble seeks to explore. I offer a brief commentary here.

 There is a consensus among Palaeolithic specialists that no houses were built before 30,000 years ago – at least in Europe. Instead, many spaces were authored to enact performances of social life, such as the horse butchery place at Boxgrove, dated to c. 500,000 years BP. One of Gamble’s most intriguing links is between the increasing brain size of hominins between 600,000 and 200,000 years BP, and their persistent use of the same places and landscapes. The slow increase in the importance of place-based and landscape-based identities for these hunter-gatherers stimulated the emergence of spatial differences between favoured and non-favoured places, and between special landscapes and other zones. The concentration of people and their objects at favoured places promoted deepening practical and emotional links which could only become deeper with ritual sanctification of practices and/or of the places themselves. But the seasonal nature of such congregations left unsolved the essential problem of Palaeolithic socialization – the missing people who were absent from their seasonal mates for most of the year. With population densities of between 0.05 and 0.005 persons per km ^2^, such absence was the norm in territories of many thousands of km ^2^ and inevitably created social issues. The appropriate Palaeolithic metaphor would be Cousin Antonio, whose absence from the home base sparked conversations such as “Where is Antonio? I haven’t seen him since last summer. I hope he’s OK.”

 Long ago, Martin Wobst suggested that open, overlapping mating networks characterized the Palaeolithic until near the end of the Last Glacial period, when locational constraints, selection for communal tasks and increasing population densities led to the emergence of new forms of mating network. In Gamblian terms, this was a new form of container – the closed mating network. The flip side of a declining momentum for seasonal mobility was the pleasure-and-pain of a more sedentary way of life, in which comments about Cousin Antonio had changed to: “What can we do about Antonio? He snores all night and sings all day.” Not only was it vital to contain Antonio in a building but the building also contained the *collections* of objects made by, or traded for, the household. The possibility that the household could eat and drink in a new state of *privacy* meant the establishment of closer, countervailing ties with those outside the private space – the neighbours – in a new form of shared hospitality, with its own social dynamic and multiplier effect. Furthermore, a building could echo, symbolize or memorialize not only associated people, animals or fish but also other significant features of the landscape, such as the trapezoidal mountain of *Treskavec* whose shape was materialized in the house-plans of Lepenski Vir. No wonder that the Neolithic soon became, in Dušan Borić’s striking phrase, “the age of building”. Gamble is correct to assert that the container- *habitus* – the house – did Neolithic persons’ thinking for them.

 What I cannot comment on directly is how increased levels of wrapping and folding in the cerebellum interacted with ‘out-of-body’ experiences to create relationships and associations between brains, people, stuff and the world. But it is a provocative metaphor, which will surely lead to new ways of conceptualizing the human condition. For this, we owe much gratitude to Clive Gamble.

Is the topic of the opinion article discussed accurately in the context of the current literature?

Yes

Are arguments sufficiently supported by evidence from the published literature?

Partly

Are all factual statements correct and adequately supported by citations?

Yes

Are the conclusions drawn balanced and justified on the basis of the presented arguments?

Partly

Reviewer Expertise:

My specialisation as primarily a ‘Neolithic’ archaeologist means that I am much more at home in the container-habitus than in an architecture without walls, although I have a good awareness of the latest trends in Palaeolithic archaeology.

I confirm that I have read this submission and believe that I have an appropriate level of expertise to confirm that it is of an acceptable scientific standard.
